# Management of cytomegalovirus corneal endotheliitis

**DOI:** 10.1186/s40662-020-00226-y

**Published:** 2021-01-14

**Authors:** Angela H. Y. Wong, Wee Nie Kua, Alvin L. Young, Kelvin H. Wan

**Affiliations:** 1 Department of Ophthalmology, Tuen Mun Hospital, Hong Kong, China; 2 Department of Ophthalmology and Visual Sciences, Prince of Wales Hospital, Hong Kong, China; 3 Department of Ophthalmology and Visual Sciences, The Chinese University of Hong Kong, Hong Kong, China

**Keywords:** Cytomegalovirus, CMV, Endotheliitis, Keratoplasty, Corneal transplant, Cornea

## Abstract

**Background:**

Cytomegalovirus (CMV) can manifest as corneal endotheliitis in immunocompetent individuals. Early diagnosis is prudent to prevent endothelial cell loss, which could ultimately lead to corneal decompensation. CMV DNA was first detected in an eye with corneal endotheliitis in 2006; since then, clinical evidence from numerous case reports and case series have accumulated.

**Main text:**

In this narrative review, we identified several drugs, including ganciclovir, valganciclovir, and their combination in oral, intravenous, intravitreal, and topical forms in different concentrations, together with the judicious use of topical steroids, have reported variable success. There has yet to be any prospective comparative study evaluating the efficacy and safety of these assorted forms of treatment; clinical evidence is based on case reports and case series. CMV endotheliitis presenting with corneal edema can masquerade as other corneal diseases and thus poses a great challenge especially in post-keratoplasty eyes. Heightened awareness is needed before and after keratoplasty to start prompt prophylaxis and treatment.

**Conclusion:**

There is no consensus on the management of CMV endotheliitis. Further studies are much needed to elucidate the optimal treatment modality, regime, and duration in the treatment and prophylaxis of CMV endotheliitis.

## Background

The herpesvirus family is a constellation of virus consisting of α (e.g., Herpes simplex), β (e.g., cytomegalovirus), γ (e.g., Epstein Barr Virus), which was postulated to have been in existence and evolved with human hosts 400 million years ago [1]. In 1954, the human cytomegalovirus (CMV) was first described by Margaret Smith [2]. It infects the majority of the population worldwide during early childhood and has a prevalence of more than 70% in the adult population [3]. CMV is a ubiquitous lymphotropic herpes virus commonly found in latent infections in the adult population; it rarely causes severe systemic disease in healthy individuals.

Khodadoust first described endotheliitis in 1982 as a specific form of corneal endothelium inflammation, characterized by localized corneal edema, keratic precipitates (KPs), mild anterior chamber reaction, and often endothelial dysfunction [4]. It was first thought to be related to an autoimmune reaction, and later found to be caused by the herpesvirus family including herpes simplex virus (HSV) [5], varicella zoster virus (VZV) [6], or occasionally Epstein–Barr virus (EBV) [7]. In 2006, Koizumi first reported corneal endotheliitis caused by CMV in healthy immunocompetent individuals [8]. Concomitant endotheliitis has been reported between CMV and HSV [9], and also between CMV and human herpesvirus (HHV-6) [10]. Unlike CMV retinitis in immunosuppressed patients, serum CMV antigen is not typically elevated, and CMV pp65 usually remains negative among those with CMV endotheliitis [11, 12]. It remains unclear why CMV retinitis is present among the immunocompromised while CMV endotheliitis is observed in the immunocompetent.

CMV is increasingly recognized as the most common virus causing corneal endotheliitis [13]. CMV endotheliitis is often presumptively misdiagnosed as herpetic eye disease [14]. Furthermore, CMV reactivation following corneal transplant presents a diagnostic dilemma as it mimics endothelial graft rejection or graft failure with generalized graft edema or endothelial cell loss [12]. This can often lead to a delay in diagnosis and treatment. If left untreated, CMV endotheliitis can lead to corneal decompensation. Endotheliitis could be exacerbated by inappropriate treatment with intensive topical steroid in the absence of antiviral treatment. Timely antiviral therapy could reverse the corneal edema and preserve endothelial cells, delaying, if not precluding the need for a corneal transplant.

There remains no consensus on the drug, dosage, duration, or modality of treatment for CMV endotheliitis. Ganciclovir and valganciclovir have been described as the drugs of choice. Different treatment regimes, including systemic ganciclovir, oral valganciclovir, topical ganciclovir, and intravitreal ganciclovir, have been used in clinical practices. Systemic ganciclovir, oral valganciclovir, and topical ganciclovir 0.15% gel are commercially available in some regions of the world. All these studied drugs are not approved by the FDA for CMV endotheliitis. The evidence drawn from the literature is limited to case reports and case series as no clinical trials are available. The purpose of this article is to review the clinical features, diagnosis, with a focus on the management of CMV endotheliitis. In vitro and in vivo responses to anti-CMV treatment, which form the basis and rationale for the proposed management for CMV endotheliitis are highlighted. The impact of CMV endotheliitis on post-keratoplasty eyes and its management are discussed.

## Main text

### Clinical features of corneal endotheliitis

CMV corneal endotheliitis can present in isolation with coin-shaped or linear KP, or in association with mild anterior chamber inflammation, ocular hypertension, or secondary glaucoma [8]. Similar clinical features are shared among other herpesvirus infections in the anterior segment. Both clinical and laboratory evidence are needed to establish the diagnosis of CMV endotheliitis. Japanese Corneal Endotheliitis Study Group proposed a diagnostic criteria combining both clinical characteristics and laboratory results, and classify the disease into typical and atypical CMV endotheliitis [11]. Patients must have a positive CMV genome in polymerase chain reaction (PCR) of aqueous humor, with a negative yield for HSV and VZV DNA, together with one of the following clinical manifestations: patients presenting with coin-shaped corneal lesions or linear KPs are classified as typical CMV endotheliitis. Those presenting with localized corneal edema with KPs are classified as atypical CMV endotheliitis, provided that two of the following signs are met: recurrent or chronic anterior uveitis; ocular hypertension or secondary glaucoma; corneal endothelial cell loss. In the study from Japanese Corneal Endotheliitis Study Group, 79 out of 106 patients (75%) were diagnosed with typical CMV endotheliitis, while the remaining were atypical CMV endotheliitis (25%) [11].

The posterior segment is typically spared in CMV endotheliitis. However, there is one reported case of an immunocompetent patient developing CMV endotheliitis, corneal graft edema and CMV retinitis, 9 months after Descemet Stripping Automated Endothelial Keratoplasty (DSAEK) [12]. The CMV genome was found in both aqueous humor and vitreous, while serum CMV antibodies and pp65 were negative. Although rare, patients with CMV infection should be monitored for CMV retinitis.

### Diagnosis of corneal endotheliitis

Aqueous tapping for CMV genome is invasive and is not without potential complications such as endophthalmitis, corneal abscess, hyphema, and injury to the lens. The sensitivity and specificity of PCR for systemic CMV infection were 80.1 and 93%, respectively [15]. Those with CMV endotheliitis were higher, reaching 90.0 and 98.7%, respectively [16]. CMV endotheliitis patients may need repeated aqueous analysis before being tested positive for the infection due to the possibility of a low viral load when the inflammation is mild [16]. Some patients may require up to 3 aqueous taps before yielding positive PCR results [17]. Clinicians should consider repeated aqueous tap if there is high clinical suspicion in cases with an initial negative PCR result.

Detectable CMV in the aqueous could be due to anterior segment inflammation or secondary reactivation by CMV. To distinguish between these entities, Kandori et al. reported that > 10^3^ copies/mL of CMV in the aqueous were associated with elevated intraocular pressure (IOP), corneal endothelial cell loss, and recurrent inflammatory episodes. Notably, IOP > 20 mmHg was most predictive of high copies of CMV (odds ratio of 18.2) and could be one of the most characteristic features of CMV endotheliitis or iridocyclitis [18]. Positive correlation was found between aqueous CMV viral load and the degree of corneal endothelial cell loss in both CMV endotheliitis and iridocyclitis eyes. This signifies the importance of aqueous viral load [19].

Several studies proposed the use of non-invasive tests as adjuvant investigations, including anterior segment optical coherence tomography (ASOCT) and confocal microscopy. Yokogawa et al. noted irregularly thickened, highly reflective endothelial cell layer on high-resolution ASOCT in CMV endotheliitis [20]. Kobayashi et al. also reported protruding structures presenting dendritic, dome-shaped, quadrangular, or saw-tooth appearance in CMV endotheliitis [21]. Hashida et al. differentiated the corneal features of CMV in contrast to HSV and VZV using ASOCT [22]. They concluded that the coin-shaped lesion in CMV endotheliitis often appeared to be quadrilateral and elliptical pattern, contrasting the small protrusion with low reflectivity in HSV endotheliitis, and the relatively larger pigmented KP in VZV endotheliitis.

Owl’s eye morphological feature has been detected on in vivo confocal microscopy (IVCM) by several reports [23–25]. The owl’s eye appearance is characterized by large cells with a prominent nucleus and high reflection, surrounded by a halo of low reflection. Peng et al. reported similar features on IVCM in an eye with culture-confirmed HSV keratitis and in 3 eyes with longstanding corneal grafts. They advocated that the reported owl’s eye feature seen on IVCM is non-specific to CMV endotheliitis [26]. There could be other reasons that could account for these findings, including concomitant CMV and HSV infection, and undiagnosed CMV infection in eyes with longstanding corneal graft. The role of ASOCT and IVCM in CMV endotheliitis diagnosis are to be evaluated, and identifying these specific imaging features is largely subjective.

### Overview of anti-CMV agents

Ganciclovir, valganciclovir, foscarnet, cidofovir, and letermovir are FDA-approved antiviral agents for CMV infections, all of them are licensed for CMV retinitis except for letermovir. They inhibit DNA polymerase and thus, its synthesis [27]. Oral valganciclovir is now the mainstay of anti-CMV treatment and have largely replaced intravenous ganciclovir. Ganciclovir is a 2′-deoxyguanosine analog, which needs phosphorylation by the CMV UL97 gene to encode viral kinases and later human kinases into ganciclovir triphosphate. Ganciclovir triphosphate is directly incorporated into viral DNA, which acts as a competitive substrate to UL54-encoded DNA polymerase, halting the viral DNA replication, and ultimately leading to apoptosis of the infected cells while sparing the non-infected cells. Ganciclovir is mainly used intravenously and maintains a high pH between 9 and 11 as suggested by the manufacturer to achieve optimal drug stability [28]. Topical ganciclovir 0.15% gel is FDA-approved for the treatment of acute herpetic keratitis. Valganciclovir, a L-valyl ester prodrug of ganciclovir, transforms into ganciclovir after hydrolyzation. It has a much better oral bioavailability of 60% compared to 6% of ganciclovir [29]. Drug resistance should be considered one of the causes for unresponsive cases, which is reported in about 10.7 and 17.2% of patients with CMV retinitis in 1-year and 2-year treatments, respectively [30]. The resistance of ganciclovir arises when there is any mutation in UL97 or UL54 [27]. There is no definition or clinical report on resistance to drug therapy in CMV endotheliitis in the published literature. For resistant CMV retinitis, second-line antiviral agents, including foscarnet and cidofovir could be considered. Letermovir has recently been approved for CMV prophylaxis in CMV seropositive hematopoietic stem cell transplant [31]. Its role has not yet been explored in ophthalmology.

### In vivo and in vitro responses to anti-CMV treatment in experiments

CMV is known to exist in a wide range of pathogenic strains with different genetic variability, growth characteristics, and different manifestations [32]. The 50% inhibitory concentration (IC50) is the inhibitory concentration of a drug to inhibit 50% of the organism’s replication. To inhibit the in vitro replication of wild-type CMV strains, IC_50_ of ganciclovir is reported to be 0.25–1.22 μg/mL [33]. No specific IC_50_ for CMV in corneal endothelial cells has been documented although an in vivo study demonstrated that high intracameral ganciclovir concentration of > 5 mg/mL could increase the risk of cell damage in cultured human corneal endothelial cell [34].

#### Intravenous ganciclovir and oral valganciclovir

Systemic anti-CMV agents, including intravenous ganciclovir and oral valganciclovir, reach intravitreal drug concentrations of 0.9–1.2 mg/L, which is similar to the minimal IC_50_ for CMV replication [13]. Intracameral concentration of ganciclovir following systemic administration has not been reported. Treatment duration for CMV retinitis typically lasts for months. The common regime includes intravenous ganciclovir 5 mg/kg twice daily for 21 days followed by maintenance therapy of 5 mg/kg/day, or oral valganciclovir 900 mg twice daily for 21 days, then reduced to 900 mg daily as maintenance [35]. However, the adverse effects of systemic ganciclovir and valganciclovir include pancytopenia, myelosuppression, nephrotoxicity, which can be life-threatening. Other adverse effects include diarrhea, nausea, fever, headache, dermatitis, insomnia, and fatigue. Complete blood count with differential counts and serum creatinine levels should be monitored 2–3 times each week during the induction period, and later once weekly during the maintenance phase for any side effects [35].

#### Topical ganciclovir 0.15% gel, topical ganciclovir 0.5 and 1% eye drops

Topical ganciclovir 0.15% gel is FDA-approved for the treatment of acute herpetic keratitis. Topical ganciclovir offers a non-invasive approach to achieve high drug concentration in the cornea or anterior chamber, which is of particular importance in patients with CMV endotheliitis. Prolonged use of topical ganciclovir 0.15% may result in blurring of vision, ocular irritation, punctate keratitis, and conjunctival hyperemia [36]. Waduthantri et al. investigated the intracameral drug concentration after the use of topical ganciclovir in 23 eyes with CMV anterior uveitis and 6 eyes with CMV endotheliitis. After a 6-week application of topical ganciclovir 0.15% 5 times per day, the mean ganciclovir concentration in human aqueous humor was 17.4 ± 30.6 ng/mL, which is below the ID_50_ for CMV replication [37].

Many attributing factors could affect the ocular pharmacokinetics of topical ganciclovir in patients with CMV endotheliitis. The lipophilic corneal epithelium acts as a rate-limiting barrier for ganciclovir, which is a polar hydrophilic compound [38]. Unlike eyes with herpetic keratitis where an epithelial defect is present, the epithelium is almost always intact in CMV endotheliitis, which hinders the penetration of ganciclovir through the epithelium. On the other hand, an edematous cornea may act as a drug depot and enhance the carrying of topical ganciclovir to the endothelium directly [39]. Ganciclovir has a high affinity to melanin and preferentially accumulates in infected cells instead of normal cells [40]. Its long intracellular half-life lasts over 24 h [41]. These factors may all contribute to the clinical effects of ganciclovir in CMV endotheliitis despite the suboptimal IC_50_ [37]. Increasing the frequency of application may increase its intracameral concentration but should be balanced with compliance and ocular surface toxicity.

Apart from 0.15% ganciclovir, other concentrations of topical ganciclovir is unavailable commercially and has to be prepared in-house. Okumura et al. investigated the physical properties of 0.5 and 1% topical ganciclovir [42]. Both 0.5 and 1% ganciclovir remained transparent up to 6 weeks across a range of temperatures. High performance liquid chromatography (HPLC) was used to measure drug concentration in different storage periods and temperatures. Both concentrations were maintained at 100% of its initial concentration at 4 °C and 25 °C for the first 6 weeks but declined gradually to 90% after 12 weeks. Their rate of decline is faster at higher temperatures. Therefore, it is preferable to store 0.5% ganciclovir solution in a refrigerator with replacement every 6 weeks.

Both osmotic pressure and pH of the drug may affect drug absorption. The osmotic pressure of the 0.5 and 1% solution is 310.33 mOsm/kg and 334.33 mOsm/kg, respectively. Likewise, the pH value were 10.7 and 10.8 immediately after preparation, and 10.1 and 10.5 at 4 °C after 12 weeks, respectively. The ganciclovir concentration of rabbit corneal endothelium reached 28.0 μg/g at 1 h and 4.3 μg/g at 3 h after instillation of a 0.5% solution; while it reached 56.3 μg/g at 1 h and 5.3 μg/g at 3 h after instillation of the 1% solution [42]. Further studies are needed to investigate the drug concentration in human eyes to optimize the regime and to address its safety issue for clinical use.

#### Intravitreal ganciclovir injection

Intravitreal injection of ganciclovir achieves higher ocular drug concentration. The aqueous humor and vitreous concentration of a single injection of intravitreal ganciclovir 200 μg in the human eye with CMV retinitis reached 0.66 μg/mL and 1.17 μg/mL, respectively after 51 h, which exceeds the minimal IC_50_ of ganciclovir for CMV replication [43]. In CMV retinitis, ganciclovir is given intravitreally 2 mg twice weekly for 3 weeks, followed by 2 mg weekly as maintenance [44]. Adverse effects including endophthalmitis, intraocular hemorrhage, vitreous humor crystallization, and retinal toxicity have been reported [45–47].

### Endotheliitis response to anti-CMV treatment in clinical cases

**Fig. 1:**
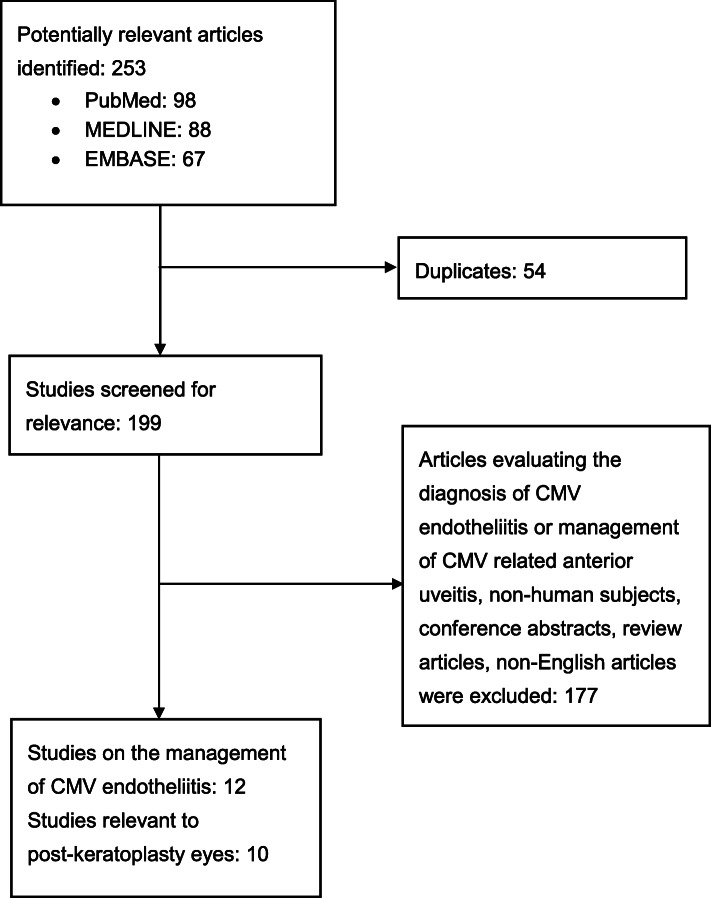
Flow chart showing selection of articles

We searched PubMed, MEDLINE, and EMBASE for relevant publications from January 1, 2000 to January 31, 2020 using keywords: “CMV”, “cytomegalovirus”, “endotheliitis”, “ganciclovir” in different and/or logic combinations. After removing 54 duplicates, we screened 199 articles for relevance. Articles that reported on diagnosing CMV endotheliitis or the management of CMV anterior uveitis as the primary outcomes were excluded. Non-clinical articles, conference abstracts, and non-English articles were also excluded. We identified 12 articles outlining the treatment of CMV endotheliitis and 10 articles relevant to the management of CMV endotheliitis in post-keratoplasty eyes (Fig. 1). There remains no consensus on the regime of anti-CMV agents in the management of CMV endotheliitis. Most studies reported clinical resolution or improvement of endotheliitis; several studies have follow-up aqueous sampling for the presence of CMV genome after treatment (Table 1). CMV endotheliitis was diagnosed based on clinical manifestations and a positive PCR result in the included studies.

**Table 1:** Study design, anti-viral regime, and steroid regime in studies on the management of CMV endotheliitis

Study	Study location	Study design	No. of eyes (patients)	Anti-viral regime	Steroid regime	Mean duration of anti-viral treatment^a^
Chee [48]	Singapore	retrospective interventional case series	12 (10)	5 patients • 6-week of intravenous ganciclovir 5 mg/kg twice daily • followed by 6 weeks of oral ganciclovir 1 g thrice daily 5 patients • 6 weeks of oral valganciclovir 900 mg twice daily • followed 6-week of oral ganciclovir 900 mg daily	topical prednisolone acetate 1% twice daily, tapered off over 3 months	12 weeks
Chiang [49]	Taiwan, China	case report	1 (1)	• oral valganciclovir 900 mg twice daily	1-week of topical prednisolone 1% 4 times a day for 1 week	4 months
Choi [50]	South Korea	case report	1 (1)	• 2 intravitreal ganciclovir injections (unspecific time interval between injections) • topical ganciclovir 0.15% every 2 h	topical prednisolone acetate 1% 4–6 times daily	N/A
Fan [51]	Taiwan, China	retrospective interventional case series	10 (9)	• topical ganciclovir 0.5% every 2 h for 1 month, then tapered to 4 times per daily	topical prednisolone acetate 1% twice daily, then tapered to once or twice daily	48 months
Hwang [52]	Taiwan, China	case report	1 (1)	• oral valganciclovir (details N/A)	topical prednisolone acetate 1% 4 times a day	2 months
Hwang [53]	Taiwan, China	retrospective interventional case series	13 (13)	• topical ganciclovir 2% every 2 h for 2 weeks, then tapered to 4 times a day	topical prednisolone acetate 1% 4 times a day for 2 weeks, then tapered to twice daily	24.5 months
Kam [54]	Hong Kong, China	retrospective interventional case series	17 (16)	13 patients • oral valganciclovir 900 mg twice daily and topical ganciclovir ointment 3 patients • topical ganciclovir ointment	prednisolone acetate 1% ophthalmic suspension	12.5 months
Koizumi [55]	Japan	retrospective, consecutive, multicenter case series.	8 (8)	• topical ganciclovir 0.15% 6 times per day	topical 0.1% fluorometholone eye drops 4 times daily	12 weeks
Pavan-Langston [56]	USA	case report	1 (1)	• topical ganciclovir 0.15% every 2 h	topical prednisolone 1% once daily	4.5 months
Wong [57]	Hong Kong, China	retrospective interventional case series	13 (11)	• 2-week loading dose of oral valganciclovir 900 mg twice daily • followed by 450 mg twice daily as maintenance	topical prednisolone acetate 1, 0.5% loteprednol etabonate eye drops, or 1% dexamethasone	ranging from 4 to 38 weeks
Yamauchi [58]	Japan	case report	1 (1)	• 5 weeks oral valganciclovir 1800 mg/dayfollowed by 900 mg/day as maintenance	topical dexamethasone 0.1% 4 times a day	3 months
Yu [59]	China	retrospective interventional case series	16 (15)	• oral ganciclovir 1000 mg/60 kg 3 times daily for 4 weeks • followed by 500 mg/60 kg 3 times daily for 8 weeks • multiple intravitreal ganciclovir injections (mean 3.47 injections) • topical ganciclovir 0.15% gel 4 times per day	topical prednisolone 0.1% 4 times daily with gradual tapering	N/A

^a^includes induction and maintenance period

*N/A* = not available

#### Systemic ganciclovir and oral valganciclovir

Chee et al. showed promising results for a 12-week systemic antiviral treatment [48]. The majority of cases achieved clinical resolution, and all subjects had undetectable CMV DNA copies after treatment. Among 12 eyes of 10 patients in the study, 5 patients were put on 6-week of intravenous ganciclovir 5 mg/kg twice daily, followed by 6 weeks of oral ganciclovir 1 g thrice daily. Five patients were given 6 weeks of oral valganciclovir 900 mg twice daily followed by 6 weeks of a reduced dose of 900 mg daily. All eyes received topical prednisolone acetate 1% twice daily tapered off over 3 months. Among the 10 eyes on antiviral treatment, endotheliitis resolved in 12 weeks in 7 eyes and was seen as early as the fourth week. Three eyes with existing bullous keratopathy remained edematous despite the resolution of the initial bullae. All eyes revealed negative CMV DNA on repeated aqueous analysis 2 weeks after treatment completion.

Following a 3-month course of oral valganciclovir, Yamauchi et al. reported a case of undetected CMV DNA on aqueous analysis at the end of treatment despite persistent bullous keratopathy as a result of endothelial cell loss [58]. Their treatment regime consisted oral valganciclovir 1800 mg/day maintained for about 5 weeks, followed by 900 mg/day as maintenance; topical dexamethasone 0.1% was used 4 times a day. CMV genome remained positive on repeated aqueous analysis 1 month after the start of oral valganciclovir, but turned negative upon completing the 3-month treatment. Chiang et al. reported promising outcomes to oral valganciclovir, which spared the eyes from requiring keratoplasty [49]. Oral valganciclovir 900 mg twice daily was initiated and maintained for 4 months. Topical prednisolone 1% was initiated at 4 times a day for 1 week. Corneal edema subsided dramatically over 2 weeks with resolved endotheliitis by 6 weeks. These 3 studies reported favorable outcomes following a 12- to 16-week course of systemic ganciclovir or valganciclovir [48, 49, 58]. Long term clinical outcomes or the presence of recurrence were not reported. Cases presenting with bullous keratopathy had poorer clinical outcomes, and thus the timing of intervention might be of prognostic value.

Hwang et al. treated an eye with CMV endotheliitis using oral valganciclovir and topical prednisolone acetate 1% four times a day [52]. The detailed regime and duration of treatment were not well described in their report. Two months after treatment, the cornea remained edematous which required penetrating keratoplasty. Aqueous humor aspiration during keratoplasty surgery revealed a positive CMV genome, but the excised corneal button was negative for CMV DNA.

Wong et al. reported a case series of 13 CMV endotheliitis or anterior uveitis eyes treated with oral valganciclovir with a mean follow up of 17.2 months [57]. A 2-week loading dose of oral valganciclovir 900 mg twice daily, followed by a twice daily maintenance dose of 450 mg. Steroid regime included topical prednisolone acetate 1, 0.5% loteprednol etabonate eye drops, or 1% dexamethasone. Among eyes with CMV endotheliitis, the duration of maintenance therapy and steroid regime varied. Two patients had no recurrence after discontinuation of maintenance therapy. Patients on 2-week and 3.5-week maintenance had no recurrence for 24 and 13 months, respectively. Bilateral CMV endotheliitis recurred in 1 patient and developed bullous keratopathy in 1 eye 4 months after stopping the 36-week maintenance valganciclovir. All cases had no follow-up aqueous analysis performed. The authors supported a 6-week course of oral ganciclovir but suggested long term maintenance therapy in other forms besides systemic anti-CMV agents.

#### Topical ganciclovir 0.15% gel, topical ganciclovir 0.5 and 2% eye drops

Treatment with topical ganciclovir 0.15% gel was first reported by Pavan-Langston et al. on an asymptomatic patient presented with granulomatous uveitis [56]. Over the ensuing 4 years, endotheliitis developed which was refractory to oral acyclovir, famciclovir or valacyclovir, and intensive topical prednisolone. Topical ganciclovir 0.15% gel was started every 2 hourly when the diagnosis of CMV endotheliitis was made in the third year. Steroid was tapered to topical prednisolone 1% once daily. Endotheliitis and uveitis resolved in 2 months while bullous keratopathy remained. After treatment cessation at 4.5 months, keratouveitis recurred but rapidly subsided 3 days after resuming intensive topical ganciclovir. Later the patient was kept on a maintenance dose of topical ganciclovir twice daily with no further recurrence. The combination of both ganciclovir and steroid in the management makes it difficult to evaluate the contributing effect of the antiviral and anti-inflammatory agents on the resolution of endotheliitis.

Koizumi et al. reported clinical resolution in the majority of the 8 CMV eyes (based on clinical manifestation and qualitative PCR) after 12-week of topical ganciclovir 0.15% gel applied 6 times per day, along with 0.1% fluorometholone eye drops 4 times daily [55]. As early as 4 weeks, 4 eyes with coin-shaped lesions resolved; all other eyes with KPs, corneal edema and anterior chamber inflammation improved. Follow up aqueous analysis at week 4 revealed negative CMV genome in 5 cases. By week 12, 6 cases had negative CMV genome in the aqueous and 5 cases achieved clinical and laboratory resolution. One eye had mild reactivation with the reappearance of CMV DNA in the aqueous, whereas corneal edema improved but persisted due to irreversible endothelial cell damage in another eye. The average endothelial cell loss after treatment was about 21.3%.

Fan et al. reported favorable outcomes in 10 eyes presented with CMV endotheliitis (based on clinical features and positive PCR result) without diffuse corneal edema treated with topical ganciclovir 0.5% eye drops every 2 h together with topical prednisolone acetate 1% twice daily [51]. Improvement was seen within 1 month with a decrease in localized corneal edema and KPs. Topical ganciclovir was tapered and was maintained at 4 times per day, along with topical prednisolone acetate 1% once or twice daily. Over the 1-year follow up period, 1 patient had recurrent endotheliitis and anterior chamber inflammation after self-discontinuation of eye drops for 1 week. Four patients developed recurrent anterior chamber inflammation without corneal lesions. Others remained well with no recurrent endotheliitis. The mean endothelial cell count was 1776 ± 834 cells/mm^2^ before treatment and 1630 ± 699 cells/mm^2^ after a mean follow-up of 48 months. This report demonstrates promising results of topical ganciclovir 0.5% with clinical resolution of endotheliitis and preservation of corneal endothelial cells, especially among those mild to moderate cases with relatively good baseline corneal function preservation.

Hwang et al. reported positive outcomes with topical ganciclovir 2% eye drops [53]. Thirteen eyes with CMV endotheliitis were prescribed topical ganciclovir 2% every 2 h, along with topical prednisolone acetate 1% 4 times a day for the first 2 weeks, followed by tapering of ganciclovir to 4 times a day and topical steroid twice daily for the next 1–2 weeks depending on the clinical condition. Responders were then put on long term maintenance therapy of topical ganciclovir 2% twice daily and topical steroid once or twice daily. All patients showed an early improvement in 2 weeks with the use of topical ganciclovir 2%. During a mean follow-up period of 24.5 months, 5 patients developed recurrence, of which 1 patient with recurrent corneal edema twice was given systemic valganciclovir 400 mg twice daily for 2 weeks for the second recurrence episode. All patients, except one with residual KPs, had a clear cornea or graft at their last follow-up. Time intervals between treatment to resolution, and between episodes of recurrence were not reported.

#### Other routes of ganciclovir added to topical ganciclovir

Choi reported one case with a combination of intravitreal and topical ganciclovir 0.15% [50]. A patient presented with endotheliitis and corneal edema, which were refractory to oral acyclovir, topical acyclovir, intensive topical prednisolone 1%, further worsened with oral prednisolone 20 mg daily. This eye was then started on topical ganciclovir 0.15% eye gel, with the discontinuation of oral steroid and intravitreal ganciclovir 2 mg was given. The corneal lesion did not improve significantly after the first injection so a second intravitreal injection was performed after an unspecific time interval between the 2 injections. Topical prednisolone acetate 1% was also reduced from 4 to 6 times daily. Corneal edema and KPs subsequently resolved 10 days after second injection. The authors did not report if repeated PCR was performed or further follow up results.

Kam et al. reported a non-comparative case series on the use of combined oral valganciclovir 900 mg twice daily and topical ganciclovir ointment in 13 patients and topical ganciclovir alone in 3 patients, along with prednisolone acetate 1% ophthalmic suspension in all patients [54]. The mean treatment period lasted for 12.5 months, but the treatment regime was not described. Overall, 15 of the 17 eyes had controlled anterior chamber inflammation. Two patients developed bullous keratopathy requiring penetrating keratoplasty.

Yu et al. reported a case series that included 16 eyes treated with intravitreal ganciclovir injection, together with topical ganciclovir 0.15% gel 4 times per day, topical prednisolone 0.1% 4 times daily with gradual taper, and oral ganciclovir 1000 mg/60 kg 3 times daily for 4 weeks followed by 500 mg/60 kg 3 times daily for 8 weeks [59]. Depending on the clinical response, intravitreal ganciclovir was given multiple times at 1-week intervals at variable doses (range: 1–3 mg). Each eye received a mean of 3.47 injections (range: 2–4 injections). Corneal edema regressed in 10 of the 16 eyes and 13 eyes had resolution of KPs. Five eyes had no improvement during the treatment period.

### CMV endotheliitis and keratoplasty

Differentiating corneal graft rejection from CMV reactivation in post-keratoplasty eyes remains to be a challenge, especially in eyes with a negative PCR aqueous sample. Treatment for immune-mediated endothelial rejection, either systemic or topical steroid, can alter the ocular immunity environment, which may exacerbate latent CMV, resulting in recurrent endotheliitis and increases the risk of graft failure [60]. Raised IOP, localized corneal edema, linear pigmented KPs, nummular or coin-shaped endothelial lesion involving both donor and recipient cornea are signs more suggestive of CMV reactivation instead of allograft rejections. An unexplained, sudden reduction in endothelial cell count should also raise concern for possible underlying CMV endotheliitis in the corneal graft. A case series by Anshu et al. identified an unexplained 52–65% endothelial cell loss 3 months after DSAEK with minimal or no anterior segment inflammation which revealed positive CMV genome retrospectively in all 4 eyes [12].

#### Anti-CMV treatment following keratoplasty

##### Oral valganciclovir

Anshu et al. reported the outcomes in 4 eyes with undiagnosed CMV endotheliitis undergoing DSAEK [12]. After noticing endotheliitis on the graft, oral steroid was discontinued and topical steroid was tapered. The individual steroid regime was not detailed but was tapered according to the duration after keratoplasty and the severity of intraocular inflammation. All patients were started on oral valganciclovir 900 mg twice daily for 6 weeks, followed by 900 mg daily for another 6 weeks. Three out of 4 eyes maintained clear graft, 2 eyes had recurrence with a mean time to recurrence 8 months after completion of treatment.

Fernández et al. used the same 12-week oral valganciclovir course on 7 DSAEK once CMV had been confirmed [61]. Topical prednisolone acetate 1% was used every 2 h in the first month, tapered over 9 months, followed by a maintenance dose of once daily. Two DSAEKs remained well during the treatment period. Two grafts failed 6 and 34 months after stopping oral valganciclovir. Another eye had primary graft failure. Two DSAEKs had recurrent endotheliitis 4 and 6 months after completion of oral valganciclovir. The two recurrent cases improved with oral valganciclovir 450 mg twice daily or topical ganciclovir 0.15% 5 times a day, respectively. Both grafts remained clear but the duration of treatment was not indicated. Maintenance therapy is recommended to minimize recurrence and graft failure in post-DSAEK eyes.

##### Combined systemic and topical ganciclovir

Dockery et al. reported an eye with repeated DMEK for corneal decompensation 3 months after the initial DMEK. During repeated DMEK, aqueous humor sampling returned positive for CMV [62]. The eye remained quiet after using both oral valganciclovir 900 mg twice daily for 2 months and topical ganciclovir gel 0.15% 5 times daily slowly tapered over 6 months. No follow-up aqueous analysis was done.

Tan et al. reported 4 cases of CMV endotheliitis (range 5–15 weeks) after uncomplicated DMEK [60]. All patients were put on oral valganciclovir 900 mg twice daily for 3 months and reduced to 450 mg twice daily, together with topical ganciclovir 0.15% 5 times daily for 6 months which was later tapered to 3 times daily. Topical prednisolone acetate 1% was started at every 3 hourly for 2 weeks and gradually tapered to once daily as maintenance at 6 months. Three cases showed clinical resolution, but the time interval was not mentioned. One case failed to respond to treatment despite later switching to IV ganciclovir and then later to IV foscarnet. Systemic therapy was stopped due to deteriorating renal function. The condition spontaneously improved with negative CMV on aqueous sampling after 2 months of topical ganciclovir 0.15% 5 times a day.

#### Anti-CMV treatment before keratoplasty

##### Combined oral and topical ganciclovir

Ideally, CMV endotheliitis should be detected in eyes before they undergo keratoplasty. Not only could treatment be initiated earlier, but it could also reduce the frequency and duration of topical steroid and its associated complication after surgery. Ang et al. detected CMV genome in the anterior chambers of 5 eyes before undergoing keratoplasty [63]. Before keratoplasty, all eyes were all treated with oral valganciclovir 900 mg twice daily for 6 weeks followed by 450 mg twice daily for another 6 weeks, together with topical ganciclovir 0.15% 5 times a day. All eyes had no endotheliitis nor anterior chamber inflammation for 6 months and undetectable CMV genome in the anterior chamber before keratoplasty. Following keratoplasty, all patients were started on topical prednisolone acetate 1% every 3 h for 1 week, thrice daily for 6 months, twice daily for 3 months, and then once daily for up to 1 year. No prophylactic anti-CMV agent was used. Two eyes had clinical resolution and undetectable CMV genome in the aqueous 6 months after treatment. The 3 CMV-positive eyes undergoing DSAEK had confirmed recurrent endotheliitis at 3, 10, and 11 months postoperatively with positive CMV DNA in the aqueous. All 3 recurrent endotheliitis cases resumed oral valganciclovir and topical ganciclovir as pre-keratoplasty treatment regime. After successful treatment, all 5 grafts remained clear over a follow-up period ranging from 13 to 44 months. However, 1 eye had graft failure 18 months later requiring repeat DSAEK; the repeated DSAEK had recurrent endotheliitis (confirmed on aqueous PCR) and eventually failed 14 months later despite oral valganciclovir and topical ganciclovir therapy. Remarkably, 2 patients with recurrence had higher viral load after surgery, ranging from a 6-fold to 2300-fold increase from before baseline (from 1.6 × 10^6^ copies to 6.0 × 10^6^ copies and from 770 copies to 1.8 × 10^6^ copies in these 2 patients). Besides viral reactivation after topical steroid use, disease transmission from donor graft with unknown CMV status could be an attributing factor. The presence of CMV DNA by PCR were present among 20% of 30 donor corneas [64]. The likelihood to acquire CMV endotheliitis through an infected donor graft and the need to screen for CMV on donor graft are questions to be addressed in future studies.

#### Anti-CMV prophylaxis after keratoplasty

Among the 3 patients who had CMV DNA detected in the aqueous at the time of keratoplasty in a case series by Hsiao et al., anti-CMV therapy was not started postoperatively until clinical features of CMV endotheliitis were evident on the corneal graft; all eyes had CMV endotheliitis postoperatively and ultimately led to graft failure [64]. Prescribing antiviral prophylaxis following keratoplasty is commonly practiced in eyes with prior history of CMV-associated anterior uveitis or endotheliitis. Performing aqueous tapping during or prior to keratoplasty to detect undiagnosed CMV endotheliitis and starting prophylaxis could be beneficial in reducing the risk of graft failure especially in regions with a high sero-prevalence of CMV. Several studies investigated the role of prophylactic anti-CMV agents after keratoplasty in eyes with a history of CMV infection [65–67].

##### Systemic and topical ganciclovir

Shimazaki et al. reported one case with repeated PKP and found positive CMV in aqueous humor during the second keratoplasty [65]. After repeated PKP, systemic ganciclovir 10 mg per day was given for 7 days, followed by topical ganciclovir 0.5% six times per day; steroid regime was not mentioned. Repeated aqueous analysis showed undetectable CMV. The graft remained clear for more than 20 months after surgery and the patient was kept on topical ganciclovir 0.5% thrice daily maintenance. Basillious et al. reported success of 2 cases receiving long term maintenance prophylactic topical ganciclovir 0.15% in preventing the recurrence of CMV-associated graft failure following DMEK [66]. Aqueous analysis at the time of surgery showed positive CMV genome in both eyes. Both patients failed to respond with persistent CMV DNA on repeated aqueous analysis following a 6- to 12-week course of oral valganciclovir, eventually leading to failed DSAEK. After receiving a DMEK for the failed DSAEK, prophylactic topical ganciclovir 0.15% was administered and titrated down to 4 times daily as long term maintenance. The use of steroid was not described. A clear DMEK without CMV recurrence was maintained for up to 29 months after surgery.

Prophylactic oral valganciclovir and long term use of topical ganciclovir 0.15% were used in a patient with bilateral CMV endotheliitis following bilateral DSAEK [67]. Before undergoing bilateral DSAEK surgery, both eyes remained clinically quiescent and CMV was negative on aqueous analysis. Prophylaxis consisting of oral valganciclovir 900 mg twice daily for 3 days before surgery was continued for 3 weeks after keratoplasty, together with topical ganciclovir 0.15% five times a day. Topical prednisolone acetate 1% was used every 3 h for 3 weeks with gradual tapering after surgery. Both eyes remained asymptomatic after keratoplasty. On a monthly routine aqueous tap screening, 1 eye was found to have a positive CMV genome 2 months after keratoplasty while on prophylaxis. Systemic valganciclovir was resumed until undetectable CMV 4 months following systemic treatment. Long term prophylactic topical ganciclovir 0.15% was given and both corneal grafts remained clear for 3 years.

##### Topical ganciclovir 0.5% eye drops

Kitazawa et al. reported favorable outcomes for long term use of topical ganciclovir 0.5% eye drops as prophylaxis in 6 eyes undergoing DSAEK [68]. Before DSAEK, all CMV-positive eyes were treated with 2-week systemic ganciclovir 5 mg/kg twice daily, topical ganciclovir 0.15% 4 to 8 times daily and fluorometholone 0.1% 2 to 4 times daily. Maintenance therapy of topical ganciclovir 0.5% 4 times daily and fluorometholone 0.1% twice daily then followed. No follow-up aqueous analysis was done, but the eyes were clinically quiet before undergoing DSAEK. Postoperatively, topical ganciclovir 0.5% 4–6 times daily was maintained for all the cases. No clinical recurrence was reported with a mean follow-up period of 40 months (range: 12–60 months). Endothelial cell loss was reported to be 26, 33, 54% at 6, 12, and 36 months, respectively, following keratoplasty.

## Discussion

The management of CMV endotheliitis is challenging and the pathomechanism of CMV endotheliitis is yet to be defined. There is no consensus on the treatment for CMV endotheliitis. The role of topical steroid in addition to antiviral treatment is debatable as successful outcomes have been reported by either antiviral alone or combinatorial treatment. Nonetheless, topical steroid should not be used alone as it can suppress cell-mediated immunity, and thus aggravate viral reactivation [12, 69]. Topical steroid combined with an antiviral is a reasonable approach to treat the subtle inflammation associated with CMV endotheliitis and low dose topical steroid with antiviral is acceptable as long term maintenance. Although there is no comparative study defining the need for topical steroid with antivirals, endothelial cell loss can occur in eyes with CMV iridocyclitis without corneal involvement [14, 19]. Furthermore, corneal immune rings develop after the resolution of CMV endotheliitis in eyes with a negative CMV PCR in the aqueous [69]. In a case series of 4 eyes, the type of KPs and CMV viral load were studied throughout treatment. The authors recommended that combined topical steroid with antiviral therapy might be indicated in CMV endotheliitis with sectoral corneal edema with and without Khodadoust line-like KPs and mutton-fat KPs. Where antiviral treatment alone could suffice in eyes with coin-shaped KPs [70], topical steroids should be used judiciously in the treatment and maintenance of CMV endotheliitis and the IOP must be monitored.

CMV-related anterior uveitis with or without ocular hypertension may be present in CMV endotheliitis as ocular tissues can be a site of CMV latency. In an acute hypertensive crisis, trabeculitis is hypothesized to be the cause of raised IOP. Whereas in long-standing cases, irreversible structural changes to the trabecular meshwork secondary to chronic inflammation could be the cause. The endotheliotropic strain of human CMV strains are not only capable of infecting corneal endothelial cells but can reorganize the actin cytoskeleton in trabecular meshwork cells, which could be one of the mechanisms of raised IOP in CMV infection [71]. CMV infection also upregulates TGF-β1, an upstream cytokine capable of inducing structural changes in human trabecular meshwork cells, which can be counteracted by the addition of corticosteroids [72]. During CMV reactivation in human mononuclear cell line THP-1 cells, the ratio of glucocorticoid receptor changes together with an increase in phosphorylation of the receptor, which can lead to glucocorticoid resistance in cases of CMV-related ulcerative colitis [73]. Further laboratory studies are needed to address whether these phenomena are observed in corneal endothelial cells and whether antiviral treatment can reverse the steroid resistance.

Despite the potential systemic adverse effect and the cost of treatment, systemic therapy including intravenous ganciclovir and oral valganciclovir is shown to achieve minimal IC_50_ for CMV replication. Promising clinical outcomes have been reported following a 12-week or 16-week course. Studies have shown that topical ganciclovir 0.15% demonstrate a favorable clinical response despite having suboptimal minimal inhibitory concentration for CMV in the aqueous humor. Topical ganciclovir 0.5 and 2% aiming at higher corneal and intracameral drug concentration showed satisfactory clinical outcomes as treatment and long term maintenance in 2 case series [51, 53]. Ganciclovir was detected in the aqueous humor of all CMV endotheliitis eyes following the application of topical ganciclovir 0.15% gel (range 24.3–691.0 ng/mL). The concentration was lower than the median effective dose in several eyes. In eyes with a low level of aqueous ganciclovir (< 50 mg/mL), there was a higher likelihood of detectable CMV DNA after treatment [55]. Based on pharmacological data using rabbit eyes, ganciclovir concentration is approximately 10 times greater in the corneal tissue than in the aqueous humor, which accounts for the observed efficacy [74]. Therefore, topical ganciclovir 0.15% should only be considered as an initial treatment option in eyes with mild inflammation without apparent corneal endothelial damage.

In other scenarios, topical ganciclovir of higher concentration alone or in combination should be the initial treatment. In CMV-positive Posner-Schlossman syndrome, following a 3-months treatment of induction therapy with topical ganciclovir 2% every 2–3 h daily and every 4 hourly as maintenance therapy, all eyes had an undetected level of CMV DNA at repeated tapping [75]. Compared to CMV-negative eyes, both groups had similar endothelial cell loss and probability of progressive endothelial cell loss. Furthermore, both groups exhibited a similar frequency of IOP spikes throughout follow up and had a comparable number of anti-glaucoma eye drops at the last follow-up. Topical ganciclovir 2% was effective for clearing the viral load, assisting the IOP control, and preserving the corneal endothelium of CMV-positive Posner-Schlossman syndrome patients. Repeated intravitreal ganciclovir might be needed to control the endotheliitis in refractory cases. Combined therapies consisting systemic, topical, and intravitreal have been explored with variable results. Eyes presenting with severe corneal edema or bullous keratopathy are less likely to regain clarity, and thus early diagnosis and treatment are advocated. It is critical to differentiate CMV reactivation from allograft rejection in eyes with keratoplasty. Pre-keratoplasty CMV endotheliitis treatment, post-keratoplasty anti-CMV prophylaxis, and cautious use of steroid may enhance graft survival. Baseline and follow-up aqueous PCR analysis could be considered to aid with the quantitative evaluation of the response to treatment.

We recognize the limitations of our review, which only included evidence from case series and case reports. Our literature search did not identify any prospective comparative studies evaluating the therapeutic management of CMV endotheliitis. Publication bias is often associated with case series and case reports as journals are more inclined to publish positive results. We speculate that those investigators who have attempted but failed to manage CMV endotheliitis with topical 0.15% ganciclovir eye drops did not publish these cases. Of note, most reports we discussed are from East Asia. For reasons yet unknown, CMV-positive corneal endotheliitis has a higher incidence among Asian patients. The generalizability of the findings to other ethnicity remains unclear due to the lack of reporting among different ethnic groups.

## Conclusions

Based on the available evidence from published case series and case reports, topical ganciclovir could be considered for the milder form of CMV endotheliitis. The choice of topical ganciclovir 0.15, 0.5% or 2% would depend on the availability of commercial preparation or compounding pharmacies formulation. For severe or refractory cases, systemic ganciclovir, intravitreal ganciclovir injection, or combination therapy may be required, but should be balanced against the potential side effects. Early diagnosis and starting treatment before undergoing corneal transplant, early use of postoperative antiviral prophylaxis, and cautious use of steroid could affect the long term survival following keratoplasty. Long term surveillance for recurrence is needed. The frequency of topical application, duration of treatment, tapering regime, and endpoint of treatment remain obfuscated, especially in post-keratoplasty eyes. Clinical trials are needed to establish a standardized treatment regime for CMV endotheliitis.

## Data Availability

Not applicable.
